# Three‐dimensional digital image construction of metaxylem vessels in root tips of *Zea mays* subsp. *mexicana *from thin transverse sections

**DOI:** 10.1002/aps3.11347

**Published:** 2020-05-26

**Authors:** Yasushi Miki, Susumu Saito, Teruo Niki, Daniel K. Gladish

**Affiliations:** ^1^ Image Processing Section MikiOn LLC 103 Ishikawa Heights, 1737 Hazama‐machi Hachioji Tokyo 193‐0941 Japan; ^2^ Department of Biology Miami University 1601 University Boulevard Hamilton Ohio 45011 USA

**Keywords:** animated 3D image, digital image reconstruction, three‐dimensional (3D) image, transverse thin‐section analysis, *Zea mays* root development

## Abstract

**Premise:**

Young plant roots share a common architecture: a central vascular cylinder surrounded by enveloping cylinders of ground and dermal tissue produced by an apical promeristem. Roots with closed apical organization can be studied to explore how ontogeny is managed. The analysis of transverse and longitudinal sections has been the most useful approach for this, but suffers from limitations. We developed a new method that utilizes digital photography of transverse sections and three‐dimensional (3D) computer virtual reconstructions to overcome the limitations of other techniques.

**Methods:**

Serial transverse sections of teosinte root tips (*Zea mays* subsp. *mexicana*) were used to construct longitudinal images, 3D images, and an animated 3D model. The high‐resolution, high‐contrast, and low‐distortion sectioning method developed previously by the authors enabled high‐quality virtual image construction with the aid of a standard laptop computer.

**Results:**

The resulting 3D images allowed greater insight into root tissue ontogeny and organization, especially specific cellular structures such as histogen layers, xylem vessels, pericycle, and meristematic initials.

**Discussion:**

This new method has advantages over confocal laser scanning microscopy and magnetic resonance imaging for visualizing anatomy, and includes a procedure to correct for sectioning distortion. An additional advantage of this method, developed to produce better knowledge about the developmental anatomy of procambium in roots, is its applicability to a wide range of anatomical subjects.

The roots of most plants have a similar anatomy, with the vascular tissues organized into a central “vascular cylinder” (stele) comprising the xylem and phloem tissue arranged in various species‐specific patterns surrounded at the outward margin by a (frequently distinct) layer of cells called the pericycle. This is surrounded by ground tissue cells of the root cortex, which is in turn contained within the root epidermis. These respective tissues are produced at the root tip by the apical meristem (Heimsch and Seago, 2008), also known as a promeristem (Clowes, 1954), a group of cells at the tip of an organ that is undifferentiated and proliferates to form the primary tissues. The roots of graminoid plants (Poaceae) such as maize (*Zea mays* L.) typically have a promeristem that has what is called “closed apical organization.” This is characterized by a distinct set of cell tiers called histogens at the apex of the root body proper that are distinctly connected to, and thought to produce, the respective tissues mentioned above in addition to the root cap. The plerome is a histogen that occupies the acropetal (distal) end of the vascular cylinders of roots with closed apical organization; the other histogens lie more distally, in the promeristems of such roots (Heimsch and Seago, 2008; Saito et al., 2019). We previously compared the promeristem structure and late‐maturing metaxylem vessel (LMX) ontogeny in a cultivar of domesticated maize (*Z. mays* ‘Honey Bantam’) with that of an undomesticated teosinte (*Z. mays* subsp. *mexicana* (Schrad.) Iltis) using a rigorous systematic analysis of serial transverse sections, and we observed noteworthy similarities and significant specific differences between these taxa with this method (Saito et al., 2019). Unfortunately, the cell‐to‐cell relationship of the initial cells of the LMXs in the vascular cylinders could not always be perfectly elucidated by observing serial transverse sections alone.

In anatomical studies, multifaceted observation strategies (use of transverse images, longitudinal images, and three‐dimensional [3D] images) should, in general, significantly improve our ability to clearly resolve problematic areas. In specific studies, however, the cell structure revealed using transverse sections has not always perfectly matched the structure revealed using longitudinal sections because they are typically made from different individual specimen organs. This has important implications for anatomical studies. If the same structures and developmental events interpreted from a longitudinal section are to be confirmed using transverse sections, ideally both must come from the same specimen, which is not possible with conventional histological techniques. A 3D reconstruction from serial sections may be an effective method for clarifying the spatial configurations of the features of a plant tissue.

The construction of 3D images may be performed using magnetic resonance imaging (MRI), which is based on radio‐frequency sections of about a 50‐μm thickness. It is a convenient system for constructing a histological atlas and/or organ model (Dhenain et al., 2001) and for observing physiological changes (Ishida et al., 2000), but the resolution level is insufficient for precise, cell‐level analyses (Staedler et al., 2013). In accordance with the improvements in computers and confocal laser scanning microscopes (CLSMs), 2D and 3D image reconstruction techniques have become more reliable. Image construction using CLSM requires the user to obtain sectional images by mechanically moving the specimen stage and performing laser “optical sectioning.” It makes possible in‐focus imaging within relatively thick specimens (by comparison to conventional light microscopy [LM] histological sectioning methods) with or without fixation of the specimen. Because MRI and CLSM data are obtained relatively non‐destructively, the accuracy of their alignments is guaranteed, and therefore procedures for image processing can be managed easily by using a computer.

However, those methods have some important limitations that prevent them from being applied widely to anatomical studies. The primary instruments that are the hallmarks of those methods are rather expensive and less widely available than photomicroscopes. Furthermore, MRI does not always provide sufficient resolution, and CLSM is restricted by interference from natural pigment molecules, fluorescence retardant treatments, and histological and/or fluorescent stain penetration. Neither MRI nor CLSM is suitable for observing fine cell structure, which currently requires a fixation technique that maintains cell ultrastructural features, appropriate staining, and the thin to ultrathin sectioning of tissue required for a good resolution of detail and contrast (Niki et al., 2019). Other methods relying on advanced technology have been successfully used for the 3D reconstruction of plant structures, such as X‐ray tomography (micro‐CT) (Staedler et al., 2013) and laser ablation technology (LAT) (Strock et al., 2019), but the latter obliterates the specimen and neither method provides as high a level of resolution as CLSM or the new method we present here.

There have been some good attempts to construct 3D images using classical thin‐sectioning methods (Levinthal and Ware, 1972; Perktold et al., 1998; Wu et al., 2011). The construction of 3D images using conventional thin sections (including ultrathin sections) has several advantages for high‐resolution imaging. It does not limit the use of stains to enhance cell structure or the use of permanent preparations that can be retained for further or alternate purposes. However, we note that the acquisition of high‐quality thin sections by conventional histological sectioning techniques requires much skill. Moreover, in our experience, obtaining and using a complete serial section series through plant tissue for anatomical analysis is limited by the need to precisely align all the images and correct for the deformation of sections derived from the embedding and cutting process, something not previously addressed.

Our objective was to develop a relatively inexpensive method for analyzing the ontogeny of tissues and cell types at high resolution, while being even more dependable and versatile than our previously published method (Saito et al., 2019). Using the high‐quality sectioning, histological preparation, and staining method developed by Niki et al. (2019) as a basis, we developed procedures to overcome the above enumerated problems and make virtual 3D images, longitudinal images, and animated 3D imagery of root tissues using photomicroscopy of serial transverse thin sections cut from resin‐embedded root tip samples with the aid of a standard laptop computer. Our specific immediate goal was to trace the ontogeny of LMX vessels; however, the method is highly adaptable to anatomical analyses of other cell and tissue types in roots and other organs.

## METHODS

### Plant materials

Cultivation methods were modified after Gladish and Niki (2000). Teosinte seeds (*Zea mays* subsp. *mexicana* from Snow Brand Seed Co. Ltd., Sapporo, Japan) were surface‐sterilized, sown in moistened vermiculite, and then placed in a constant 25°C in a continuously dark growth chamber for four days.

### Preparation of thin sections for LM

The procedures used for LM were modified from Niki et al. (1995, 2015). Root tip segments were excised from the selected roots (1.0–1.5 cm), immediately immersed in 4% (w/v) paraformaldehyde in 0.1 M phosphate buffer, and gently shaken overnight at room temperature. Following fixation, the specimens were rinsed in buffer, dehydrated in an ethanol series, embedded in Technovit 7100 resin (Heraeus Kulzer GmbH, Wehrheim, Germany), and serial sections were made transversely at a thickness of 1 μm on a Reichert‐Nissei UCT ultramicrotome (Leica Ltd., Tokyo, Japan).

RNase‐treated sections were prepared for high‐resolution and high‐contrast LM micrographs according to Niki et al. (2019) as follows. The sections were mounted on glass slides and treated with ribonuclease A (RNase; Sigma‐Aldrich, St. Louis, Missouri, USA) at 60 μg/300 μL 0.05 M phosphate buffer solution (pH 7.2), which removed RNAs that would otherwise be heavily stained because of their cytosolic abundance (Sugiura and Takeda, 2000) and obscure the image. The slides were kept in an incubation box at 100% humidity and 37°C for 4 h. After washing with distilled water, the sections were stained with 1% (w/v) toluidine blue O (TB) (Electron Microscopy Sciences, Hatfield, Pennsylvania, USA) at 45°C for 5 min and rinsed with distilled water.

### Acquisition of photographic images of serial sections

The sections were viewed with a Leica DMLB light microscope (Leica Microsystems GmbH, Wetzlar, Germany) equipped with TU Plan Fluor 50× ∞/0 lenses (Nikon, Tokyo, Japan) and photographed with a Canon EOS 5D Mark II digital camera (Canon Inc., Tokyo, Japan). The original image size was 5616 × 3744 pixels, which corresponds to 270 × 180 μm at 50× magnification. Photoshop Elements 2018 (Adobe, San Jose, California, USA) and Mathematica version 11 (Wolfram Research, Champaign, Illinois, USA) were used for image processing and numerical computations, respectively.

**Figure 1:**
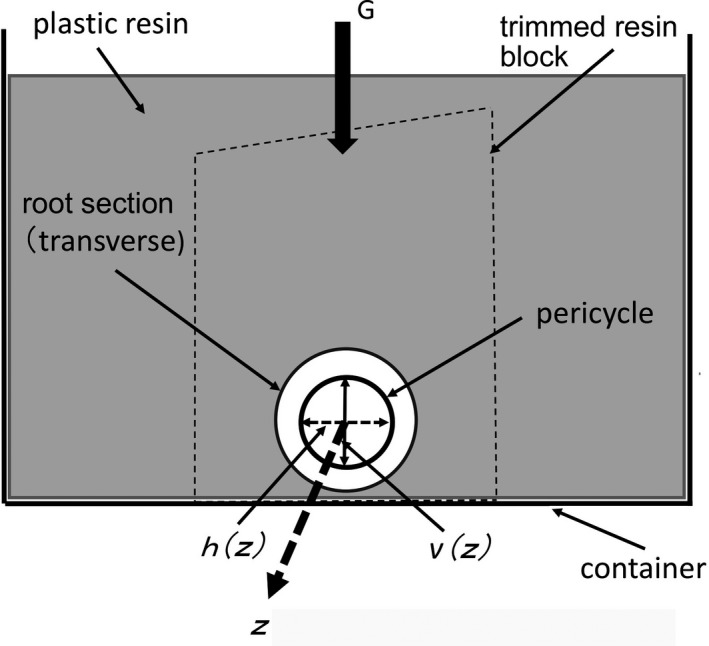
Schematic drawing of embedding a sample in resin and trimming sections. In the inner circle (pericycle of the root), the solid arrow represents the pericycle diameter parallel to G, while the broken arrow is perpendicular to G. G: gravity vector; *z* is the longitudinal direction of the root, and *z*,* v(z)*, and *h(z)* represent the coordinate axes of the 3D virtual matrix used to construct a virtual cuboid.

In order to successively arrange the photographs of all segments to correspond to the original root, the images were taken in a coordinate system which was defined as follows. As a root immersed in a plastic resin sank down (because a root is denser than the resin) to near the flat bottom of a container placed in a horizontal plane, the vertical direction to the flat bottom through the center of the root was defined as the *v*‐axis (the gravitation direction). The *z*‐axis passed through the center of the root segment parallel to the root's natural longitudinal axis, and the *h*‐axis passed through the intersection of the *v*‐ and *z*‐axes normal to both and parallel to the container bottom (Fig. 1).

**Figure 2:**
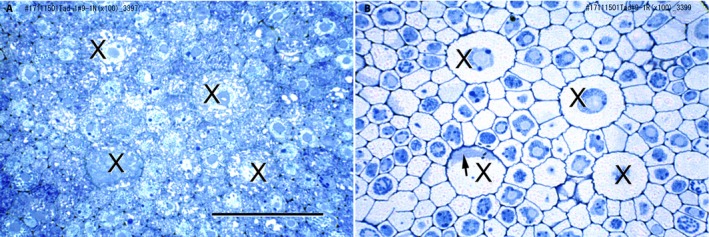
Toluidine blue–stained transverse sections of the vascular cylinder of teosinte root tips. (A) Untreated section. (B) RNase‐treated section. Arrow: portion of transverse cell end wall; X: late‐maturing metaxylem vessel (LMX). Scale bar = 50 μm.

**Figure 3:**
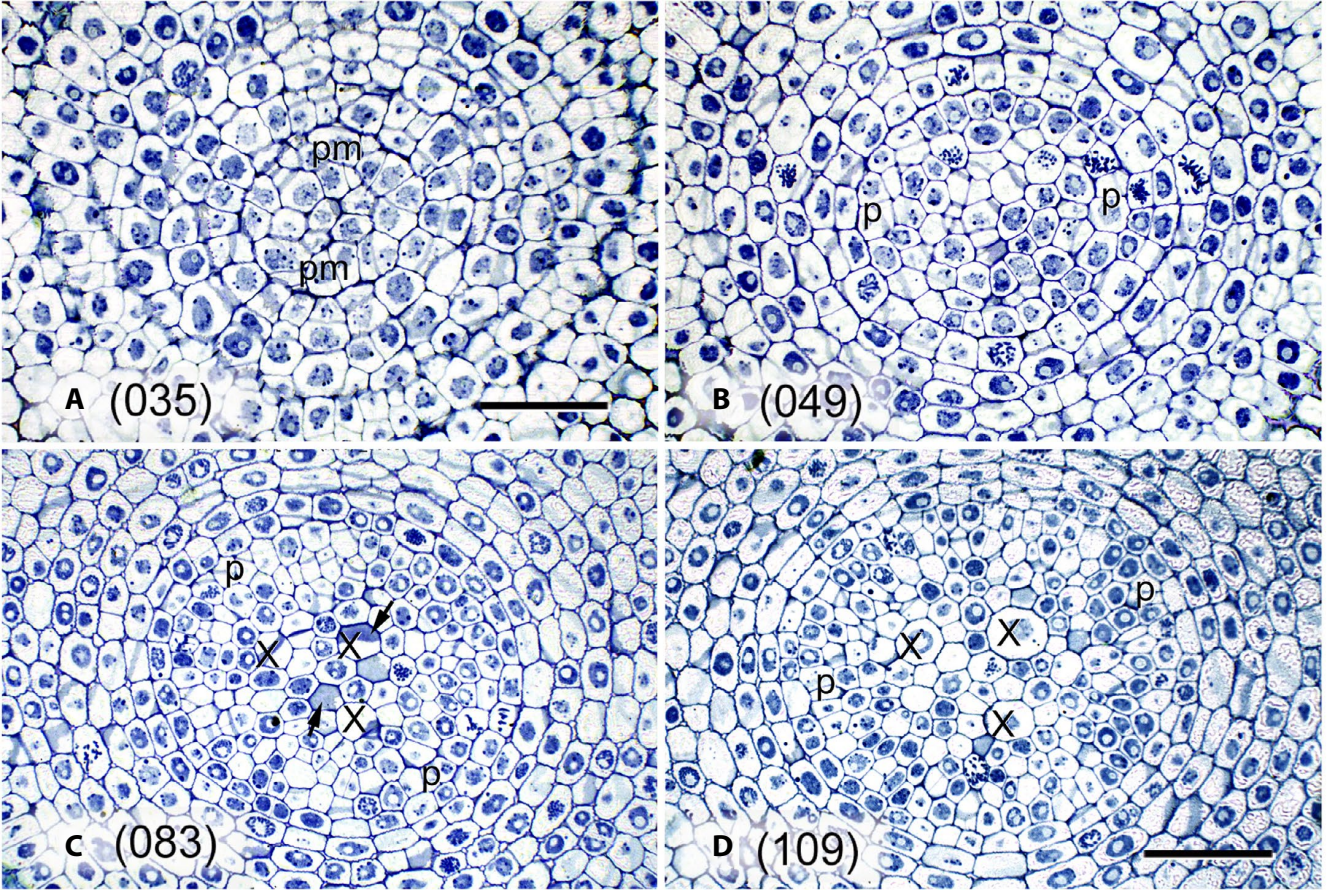
Selected enlarged images of (A) section 035, showing the plerome at the center; (B) section 049; (C) section 083; and (D) section 109 from the photo files (Appendix S1). Arrow: portion of transverse cell end wall; pm: plerome margin cells, the initials that divide to produce the pericycle; p: pericycle; X: late‐maturing metaxylem vessel (LMX). Scale bar = 50 μm.

TB stains carbohydrates and nucleic acids strongly, so cell walls, chromatin, and cytoplasm stain well. Consequently, the demarcation of cell walls from cytoplasm is sometimes difficult. The cells of root tips are typically cytoplasmically dense and stain heavily with TB due to their high RNA content. For this reason, RNase treatments were performed on most sections used for this study (Niki et al., 2019; Saito et al., 2019). Figure 2 shows micrographs of adjacent transverse sections of the central part of the same root tip; the section in Fig. 2A was stained with TB and in Fig. 2B was stained with TB following an RNase treatment. LMX and the other cells in Fig. 2B are more distinct than those in Fig. 2A. By eliminating RNA, the RNase treatment allowed the cell walls to be more sharply defined, which facilitated the alignment of the sequential sections. Appendix S1 shows photographs of 127 successive transverse sections used in the present study, and Fig. 3 shows enlarged micrographs that were selected from the 127 images shown in Appendix S1 to illustrate the initial appearance of the micrographs before digital image processing.

### Image processing

Images were processed using the pencil, paint bucket, and resolution adjustment tools in Photoshop Elements version 16.0 (Adobe), and plots were computed and drawn using the ListPlot[] function in Mathematica version 11 (Wolfram Research) running on a MacBook laptop computer equipped with MacOS Mojave (Apple, Cupertino, California, USA). The resolution of section images (Appendix S1) was reduced to 1404 × 936 pixels because it was sufficient for the present purpose. The pixel size is given as 270 (mm) / 1404 = 0.192 (μm). Because some of the images were not usable due to sectioning defects (Appendix S1), for the purpose of processing, the image preceding a damaged section was repeated at those places (numbers 021, 024, 039, 058, 069, 096, 098, 104, 115, 121, and 124 in Appendix S1) to preserve the original tissue dimensions.

The first image preprocessing step was to erase the cell nuclei in the central major part of each image (vascular cylinder and several surrounding cell layers) to emphasize the cell walls. The second step was to colorize specific cells (the central cells of the plerome and its marginal initials that produce the pericycle, the pericycle layer, vascular initials, and the resulting LMX) in each image to distinguish them from other cells (LMX 1: red/red‐orange, LMX 2: yellow/orange, LMX 3: emerald‐green/yellow‐green, pericycle: light green, etc.), and longitudinally adjacent cells in each LMX file were given different but similar alternating colors (e.g., red/red‐orange) (Appendix S2). The end walls of the cells in the longitudinal files were determined from their shadow‐like appearance in the images of the transverse sections (Figs. 2B and 3C; cf. Fig. 2, Niki et al., 2019).

**Figure 4:**
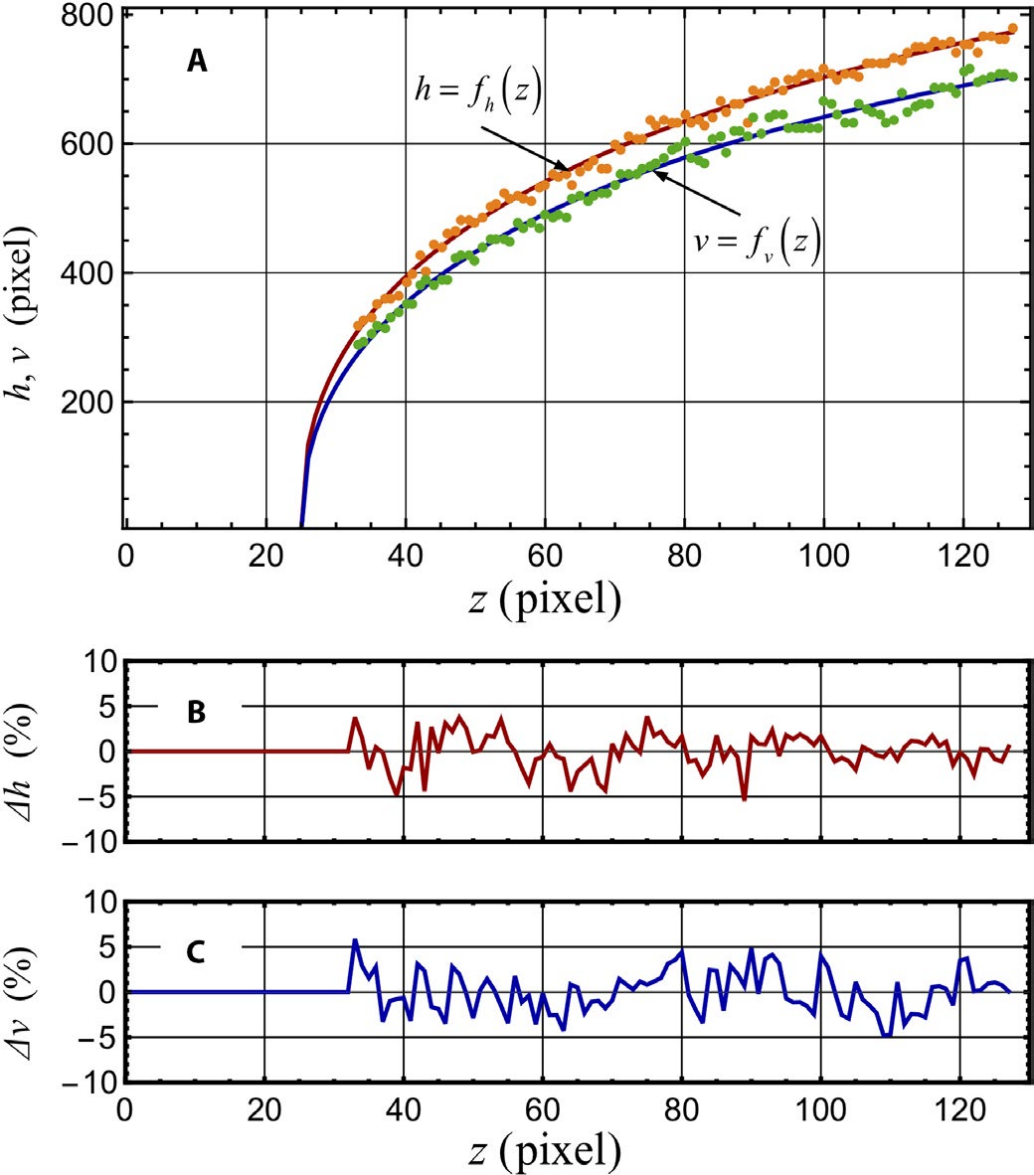
(A) Diameters of a vascular cylinder at its tip (procambium, delimited by the root's pericycle) measured from the layered serial sections in Appendix S1. Dots represent measured diameters of horizontal and vertical directions, *h*(*z*) and *v*(*z*) (orange and green, respectively). Lines represent fitted curves to the measured diameters (red and blue, respectively). The *z*‐axis represents the longitudinal distance of root from the apex of the pericycle where it originates (the plerome margin) in micrometers with an offset of +25 μm. The *z*‐axis values also indicate the section number of 1‐μm‐thick sections in Appendix S1 and the pixel number along the *z*‐axis. The horizontal and vertical axes (*h*‐, *v*‐axis) describe the diameter of the vascular cylinder and are shown in pixels (1 pixel = 0.192 μm). (B) and (C) show relative deviations (%) between the measured data and the fitted curves. *Δh* = (*h*(*z*) – *f*_*h*_(*z*)) / *f*_*h*_(*z*), *Δv* = (*v*(*z*) – *f*_*v*_(*z*)) / *f*_*v*_(*z*), respectively.

Before all the layers of images are aligned and stacked to construct a 3D object, distortions of each section that may have occurred in the sectioning process must be estimated in the third preprocessing step. Because the diameter of each vascular cylinder, defined by its pericycle, appeared to be well‐ordered and to increase gradually with each increasing layer number, it could be used as an index of distortion. However, the diameters along the horizontal and vertical axes (Fig. 1) differed slightly from each other, so the analysis of each was performed separately. In Fig. 4A, the orange dots and green dots show the measured diameters for the horizontal and vertical directions, respectively. The curves shown in red and blue are the fitted curves for the data of either direction and are given as:

(1) $$f_{h}\left(z\right)=25.0+74.0872\;z^{1/3}+62.2143\;z^{1/2}-1.97588\;z,$$

(2) $$f_{v}\left(z\right)=25.0+40.7417\;z^{1/3}+74.1939\;z^{1/2}-2.3059\;z,$$

respectively. The variable *z* represents the longitudinal distance in micrometers along the root axis from the acropetal face of the plerome with an offset of +25 μm to account for the sections before the plerome face in the series shown in Appendix S1, and also indicates the number of 1‐μm‐thick serial transverse sections and the pixel number along the *z*‐axis. The relative deviations between the measured data and the fitted curves for the horizontal and vertical directions, Δ*h* and Δ*v*, are given as

(3) $$\Delta h=\frac{h\left(z\right)-f_{h}\left(z\right)}{f_{h}\left(z\right)},$$

(4) $$\Delta v=\frac{v\left(z\right)-f_{v}\left(z\right)}{f_{v}\left(z\right)},$$

respectively, which are shown in Fig. 4B and 4C, and they are within 5% of the pericycle diameter.

If any distortion of the section was perceived, the images of each section were corrected by applying a scale operation of Photoshop Elements, and finally all the image layers were aligned precisely so that the cells in the adjacent layers would overlap each other well.

## RESULTS

### Construction of longitudinal images using transverse sections

A 3D virtual object was obtained by stacking the corrected layers, and its central portion was “extracted” to form a cuboid of 800 (width) × 800 (depth) × 127 (height) pixels. By defining cutting planes through the cuboid, any sectional view can be computed to investigate the structures of the cell and tissue components (e.g., LMX).

**Figure 5:**
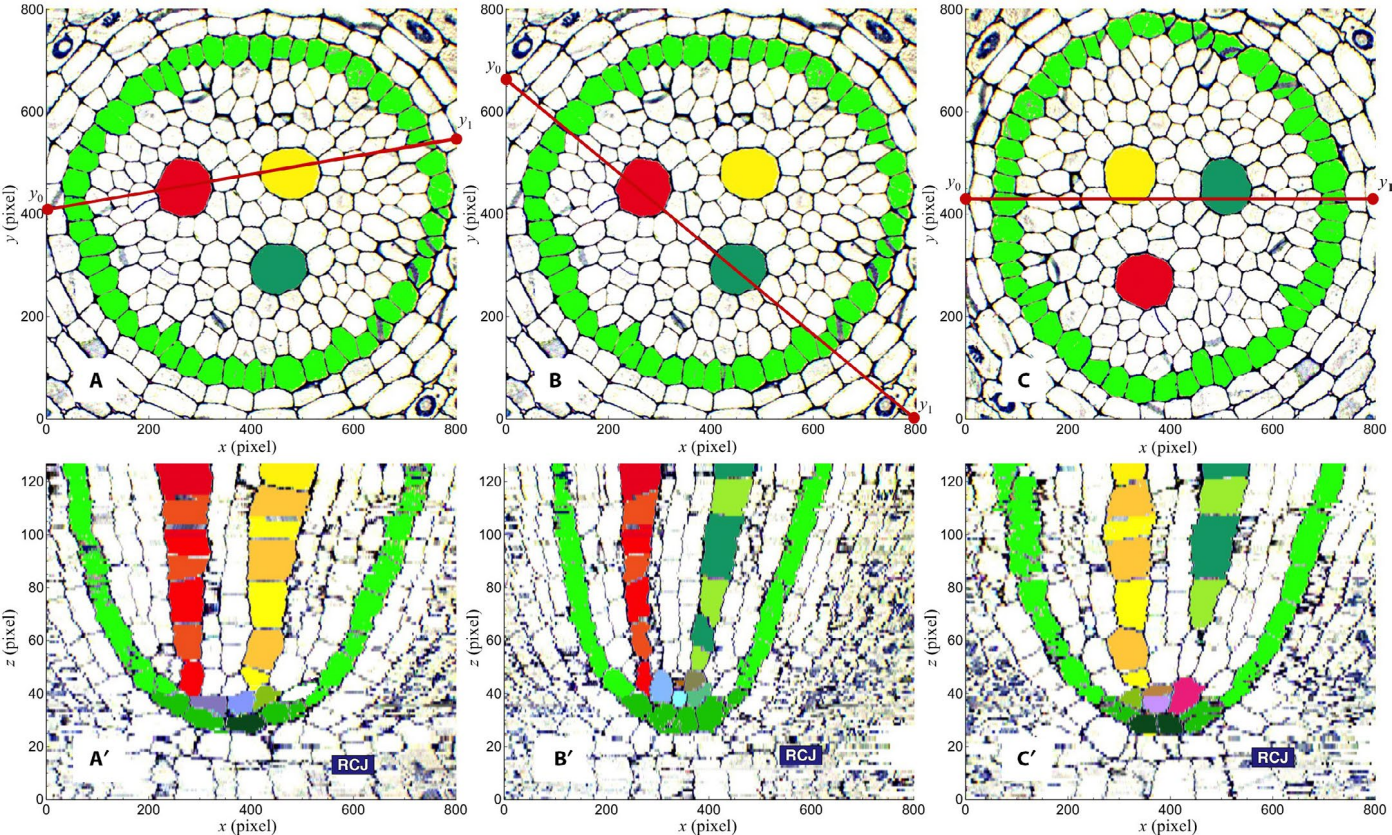
Plan views of the cuboid (A, B, C) and corresponding “virtual” longitudinal sectional views along the line segments indicated by red lines (from Eq. 5). (A): *y*_0_ = 410, *y*_1_ = 550; (B): *y*_0_ = 660, *y*_1_ = 0; and (C): *y*_0_ = 430, *y*_1_ = 430. The object shown in (C) is rotated counterclockwise by 90° with respect to (A). Specific cells have separate colors (late‐maturing metaxylem vessel [LMX] 1: red/red‐orange; LMX 2: yellow/orange; LMX 3: emerald‐green/yellow‐green; pericycle cells: light green; plerome margin initials: green; plerome central cells: dark green). Alternating cells in each LMX cell file have been given different but similar colors (e.g., red/red‐orange). Other colors are cells of the vascular initials layer. Each plan view (A, B, C) consists of 800 × 800 pixels, and the pixel size is given as 270 (mm) / 1404 = 0.192 (μm). The pixel size of each sectional view (A′, B′, C′) along the *z*‐axis is 1 μm. The root cap junction is indicated as RCJ in each sectional view. The upper panels (A, B, C) show the location of the longitudinal view transecting the cuboid shown in the corresponding panel below it (A′, B′, C′).

The upper panels of Fig. 5 (A–C) show the location of the longitudinal view transecting the cuboid that is shown in the corresponding panel below it (Fig. 5A′–C′). Each image consists of 800 × 800 pixels, and the pixel size is 0.192 (μm), as described above. Let (*x*,* y*) be the point on the image of each layer of the cuboid and

(5) $$y=\frac{y_{1}-y_{0}}{800}x+y_{0}\;\left(0\leq z\leq 800\right)$$

be the line segment indicating a vertical cutting plane, where *y*_0_ is the *y* coordinate at *x* = 0 (i.e., the left end of the line segment) and *y*_1_ is the *y* coordinate at *x* = 800 (i.e., the right end thereof). Pixels on this line segment at each section were extracted and set to a corresponding sectional layer in order to construct a longitudinal section image. If that cuboid(*x, y, z*) is the cuboid in question, the line element of the section at layer *z* will be given as

(6) cuboid(x,round(y),z)(1≤x≤800),

where round() means the round‐off function and *y* is given as Eq. (5).

The lower panels of Fig. 5 (A′–C′) show the longitudinal section views generated along the line segments shown in red in the corresponding upper panels with (A) *y*_0_ = 410, *y*_1_ = 550; (B) *y*_0_ = 660, *y*_1_ = 0; and (C) *y*_0_ = 430, *y*_1_ = 430, respectively. Virtual object (C) was rotated counterclockwise by 90° with respect to (A). Any other sectional view is available if *y*_0_ and *y*_1_ are chosen between 0 and 800.

Each longitudinal cross‐sectional image (Fig. 5A′–C′) shows the cell organization of two LMX together with the pericycle and surrounding cells.

### 3D presentation of LMX in a vascular cylinder

To investigate the 3D structure of the LMX, the cuboid described above was further reduced to the central portion of the vascular cylinder of size 360 (width) × 360 (depth) × 60 (height). In total 60 sections (numbers 021–080) from the original section set were used, and the dimensions of each image were 360 × 360 pixels (Appendix S2). Individual cells in these layers could be traced to the neighboring layers, and the transverse end‐walls of the cells were determined from “shadow” patterns, which appear, for example, in the yellow cell of section numbers 049 and 050.

Regions of the colored LMX and their initial cells could be revealed in the 3D domain by making all regions in the cuboid, except for those cell types, transparent. Each 3D element of the cuboid (“voxel”) consists of color information, i.e., cuboid(*x*,* y*,* z*) = (*r, g, b, a*), where (*r*,* g*,* b*) are the RGB colors (0 ≤ *r,g,b* ≤ 1) and *a* represents the opacity. If *a* is set to zero, the voxel becomes transparent.

**Figure 6:**
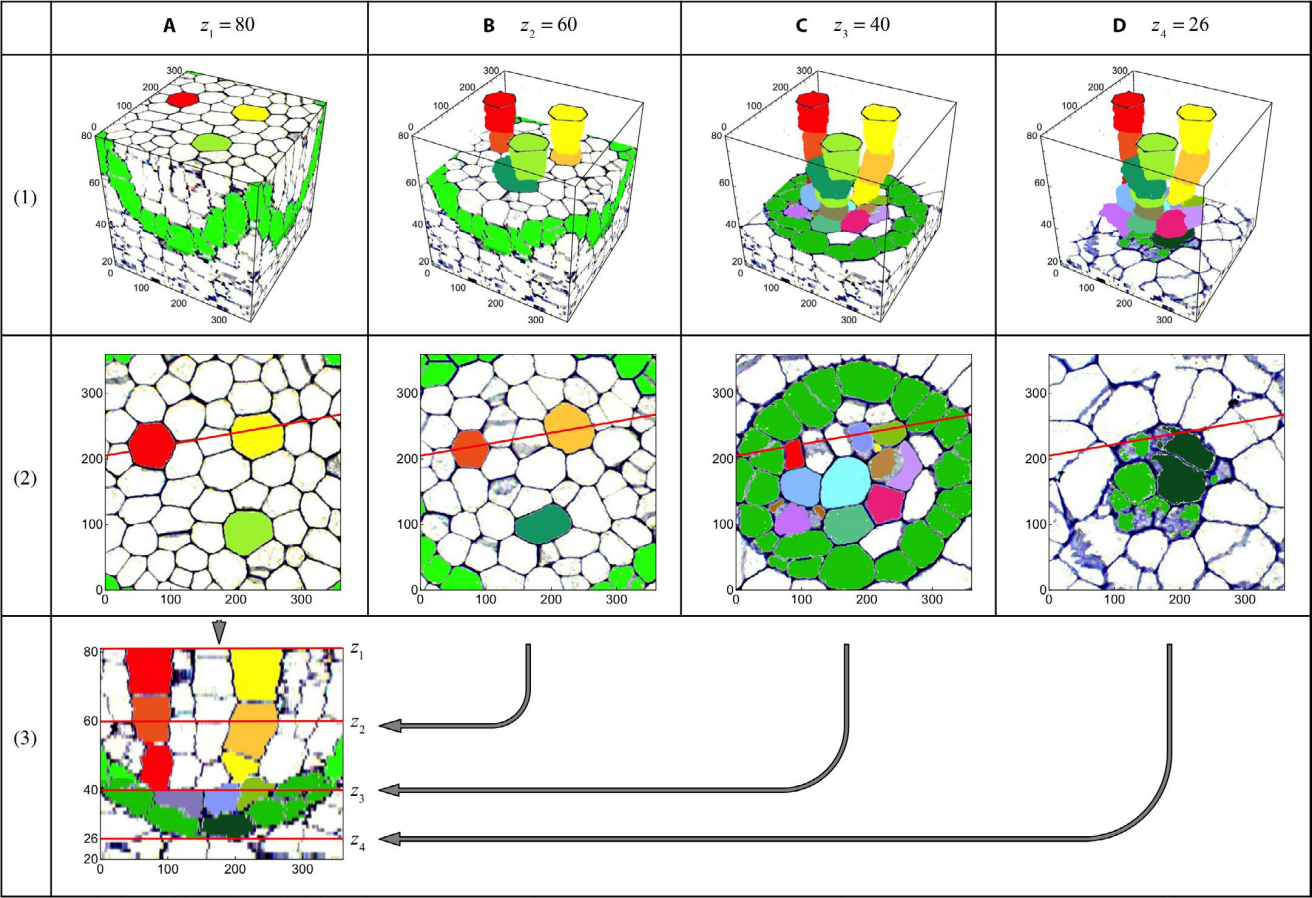
3D extraction procedure for revealing the late‐maturing metaxylem vessel (LMX) structures. (A1) Cuboid (360 × 360 × 60 pixels) constructed from the data shown in Appendix S2. The pixel sizes along the horizontal axes and the vertical axis are 0.192 (μm) and 1 (μm), respectively. (B1–D1) Process of 3D extractions sequentially from the top layer *z*_1_ = 80 μm from the root cap junction (RCJ) down to the layer *z*_2_ = 60 μm, *z*_3_ = 40 μm, and *z*_4_ = 26 μm from the RJC in order. (A2–D2) The transverse layer image of the cuboid at the corresponding respective *z* level. The red line segments in row 2 show the location of the cutting plane of the virtual longitudinal section (A3) where it intersects the image. The corresponding transverse layers *z*_2_ to *z*_4_ are shown with red lines in A3 and are indicated with arrows.

Fig. 6A1–D1 shows the sequential stages of the 3D extraction procedure for the LMX, where (A1) is the cuboid (360 × 360 × 60) constructed from the data shown in Appendix S2, and (B1–D1) are the 3D extractions from the cuboid top layer *z*_1_ = 80 down to the layer *z*_2_ = 60, *z*_3_ = 40, and *z*_4_ = 26, respectively.

**Figure 7:**
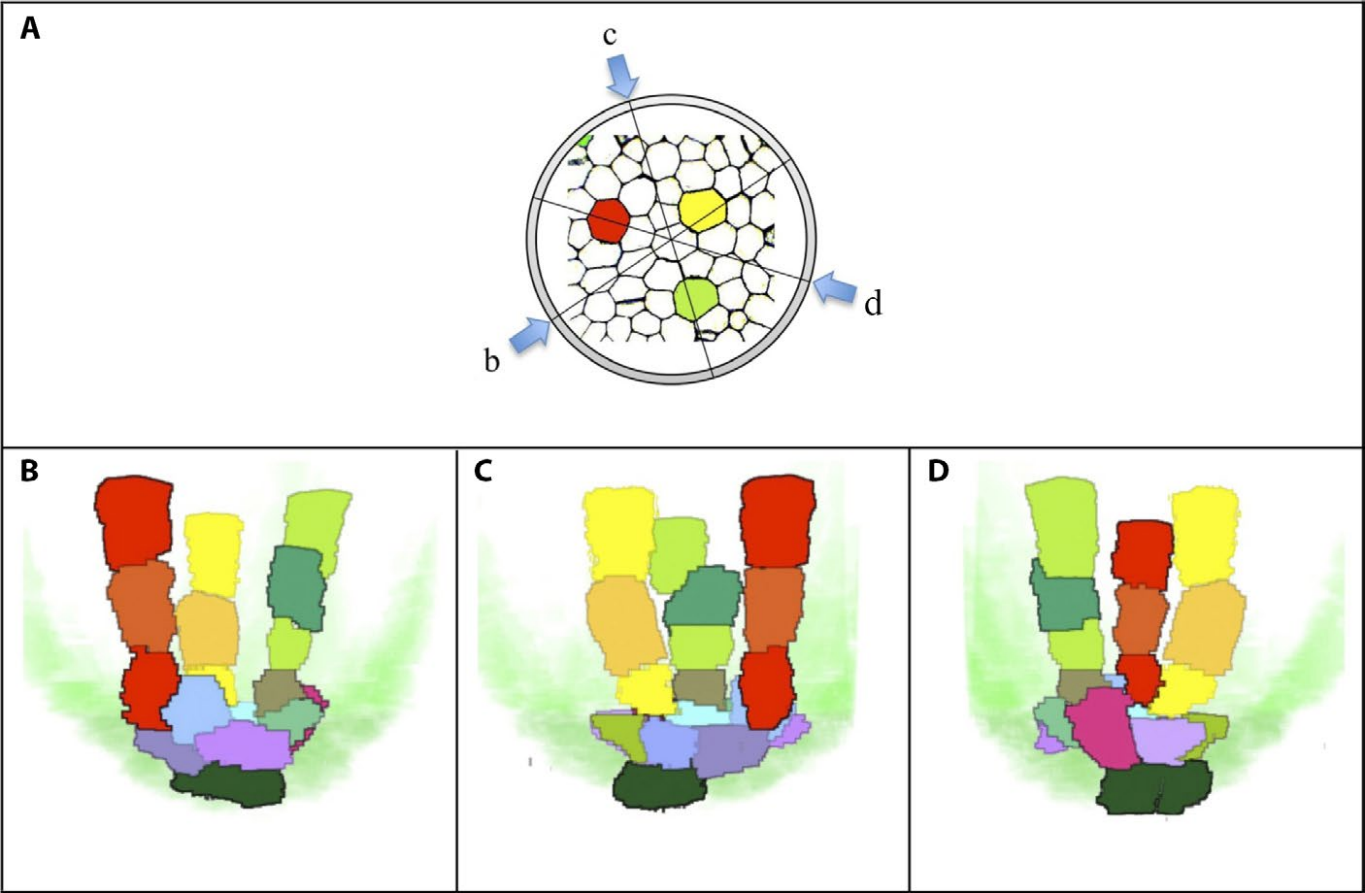
Visualization of the 3D interpretation of three late‐maturing metaxylem vessels (LMXs) and their initial cells viewed from three different angles. (A) Plan view of 3D reconstruction. (B–D) Corresponding sectional views. The pericycle and plerome are shown in translucent green and dark green colors, respectively.

Fig. 6A2–D2 shows the transverse layer image of the cuboid at the corresponding *z* levels. The red line segment defined a cutting plane corresponding to the longitudinal section of Fig. 6A3, and the red lines *z*_2_ to *z*_4_ in this figure corresponded to the layers shown in Fig. 6B2–D2, respectively. Thus, the 3D object showing the LMX vessels together with the initial cells was produced. The virtual object can be viewed in any orientation, some of which are shown in Fig. 7, and an animated representation is also provided (Video S1).

## DISCUSSION

High‐quality images are essential for image processing by computer and necessarily must consist of high‐resolution, high‐contrast, and low‐distortion micrographs of physical sections. Many types of microscopes, including electron microscopes, have been developed and improved for morphological and anatomical studies. Sampling methods have also been continuously improved (Haseloff, 2003).

There have been attempts to construct 3D images using MRI, but the sections generated using this technology are relatively thick (typically about 50 μm; Dhenain et al., 2001; Sharpe, 2003; Majka and Wójcik, 2016) and resolution is limited to >25 μm (Staedler et al., 2013). This very expensive tool would therefore only be appropriate for observing relatively large‐scale tissue or organ structures. Other methods for constructing anatomical 3D images include X‐ray tomography (also known as micro‐CT) and LAT. The former produces reasonably good results at a fairly high resolution (3 μm) following the infiltration of an appropriate “contrast agent” such as phosphotungstate and critical‐point drying (Staedler et al., 2013). The latter, LAT, has the advantage of high throughput, but is limited in resolution to >0.1 mm (Strock et al., 2019), so it is even less useful for studying fine anatomical details than MRI and has the disadvantage of destroying and discarding the sample as it proceeds. Wu et al. (2011) produced an excellent 3D study of root anatomy using LM, image processing, and computer‐aided 3D reconstruction, revealing useful information about vascular anatomy and the spatial distribution of lateral roots in winter wheat, but at a much lower resolution than the present study.

CLSM is a noteworthy tool for modern research. It enables the in‐focus imaging of relatively thick specimens by optical laser sectioning, but cannot exceed the optical resolution limits imposed by the subject. On the other hand, it is very useful for the analysis of some physiological functions because live tissue may sometimes be used, and it is useful for constructing 3D images of various styles by computer without physical sectioning (Fischer et al., 2011). However, in our experience (unpublished data), CLSM is limited by the use of various stains, fluorescent retardants, interference by natural pigments, and the use of traditional histological methods to make slides permanent (e.g., adhesives and glass cover slips). In practice, the intrinsic properties of CLSM mean it is of limited use for imaging live or uncleared plant samples because the imaging depth is restricted to 30–50 μm, the depth of only a few layers of cells, which is much lower than the theoretical limits of modern confocal microscopes, and the image quality is not very good (Haseloff, 2003; Truernit et al., 2008; Warner et al., 2014).

In the past few years, new methods have been developed to improve CLSM (Bougourd et al., 2000; Haseloff, 2003; Fischer et al., 2011). By clearing the tissue and using particular stains, such as analine blue, pseudo‐Schiff propidium iodide, or Calcofluor, researchers have successfully used CLSM to reveal tissue architectures to the level of the cell and to construct 3D images of the *Arabidopsis thaliana* (L.) Heynh. embryo and tissues (Bougourd et al., 2000; Truernit et al., 2008; Wuyts et al., 2010), as well as *Zea* L., *Pisum* L., *Nicotiana* L., *Medicago* L. (Warner et al., 2014), and *Bowenia* Hook. (Coiro and Pott, 2017). However, clearing agents such as chloral hydrate, fluorescent stains such as Calcofluor White MR2, and tagged antibody probes require a long time (5 d) to penetrate the plant tissue (Warner et al., 2014). Furthermore, although cleared tissues can be resolved to ~0.5 μm (Truernit et al., 2008; Warner et al., 2014) at a tissue depth of up to 200 μm (Haseloff, 2003), the clearing treatment results in the extraction of many cellular constituents and the alteration or disruption of subcellular structure, which limits CLSM to mapping tissue architecture. Our method has the virtue of allowing the conservation of the well‐preserved cell ultrastructure contained in the original high‐contrast thin sections, facilitating its reanalysis using other methods. In fact, with the use of ∞/0 objective lenses, which do not require cover slips on slides, the section can be stained with TB, photographed, and then treated with RNase, so that the original cytoplasmic detail can be retained photographically before image processing for 3D reconstruction (Niki et al., 2019).

For the present study, 127 micrographic image files made from transverse serial sections cut from resin‐embedded root segments were used to construct longitudinal images and 3D images. Because the sections were 1 μm thick, the spatial resolution was estimated to be 0.5 μm based on the resolving power of enlarging the digital pixel image. This value is close to the theoretical resolving power of a light microscope (0.2–0.3 μm) and can be dependably produced by our sectioning method (Niki et al., 2019). The contrast of the images of our sections was clear and high (Fig. 2B), which facilitated image processing by a computer.

The evaluation of the distortion of sections has been a matter of concern, but no articles seem to have been published on this issue. Here, we examined the distortion of the obtained sections from the viewpoint of the fluctuation of the contour size of the pericycle in a given root (Fig. 4). The axes *v* and *h* in the sections (Fig. 1) are the direction of gravity (G) and the horizontal direction at the time of resin polymerization, respectively. G may contribute to slight but consistently smaller *v* values in each section. G may also favor the settling of the specimen in an orientation so that *v* < *h* if there is natural asymmetry in the specimen. Furthermore, the direction of cutting by the glass knife of a microtome is parallel to the plane formed by the *h* and *v* axes, and cut in the direction of the G vector. However, the distortion due to the shear of sections used in the present work was limited to a small fluctuation under 5% (Fig. 4), and showed no relation to the direction of *v* and *h* (Figs. 1, 4). These data showed that the distortion of sections embedded in Technovit 7100 resin due to sectioning was small, so it did not interfere with the image alignment for image processing with a computer.

The distortion of the pericycle can be considered as that of the section itself, and, as previously stated, the relative fluctuation of distortions was found to lie within 5% of pericycle diameter (Fig. 4). Furthermore, a method for correcting the distortion was made possible by scaling the images in accordance with the amount of the relative errors between the measured data and the fitted curves. Thus, the set of section images was made suitable for image processing.

During the sectioning of blocks of resin‐embedded tissue, some failures occur even at the hands of skillful specialists (described above), but this physical sectioning method has several strengths. The method allows the uniform resolution of cell and tissue detail throughout the sample regardless of the tissue depth, and is not limited by the presence of stains or fluorescence; in fact, the method is enhanced by these. Furthermore, the original physical sections can be saved as permanent preparations for decades, and can therefore be available for the later confirmation of images or reimaging in the future. The image processing in this work was completely undertaken using a regular personal laptop computer and general‐purpose, widely available software.

The aim of the present work was to develop and fine‐tune a method to facilitate deep‐tissue imaging and virtual 3D reconstruction of plant tissue from transverse serial sections as part of a larger project to reanalyze the procambium ontogeny in detail. Although the methods presented in this paper to obtain serial transverse sections from blocks of resin‐embedded tissue are laborious and require special skills, the resulting set of serial section images is versatile and can provide fine longitudinal 2D and 3D images with the aid of a personal computer equipped with appropriate, readily available software. Furthermore, there is no reason this approach may not be used for other tissues or organs in plants—or for other types of organisms, for that matter.

## AUTHOR CONTRIBUTIONS

Y.M. designed the digital protocols that permitted the conversion of photomicrographic data into digital data suitable for analyzing tissue development in three dimensions, and also performed the data analysis and contributed to the writing of the manuscript. S.S. performed the photomicroscopy and data interpretation. T.N. used novel histological methods to prepare the plant material for microscopy, performed the data interpretation, and contributed to the manuscript. D.K.G. performed the data interpretation and prepared the manuscript for publication.

## Supporting information


**APPENDIX S1.** Micrographs of 127 RNase‐treated, toluidine blue–stained transverse serial sections from a single teosinte root tip. Each section is 5616 × 3744 pixels, corresponding to 270 × 180 μm at magnification of 50×.

**APPENDIX S2.** Layers of the cuboid produced from 60 transverse sections (sections 021 to 080 of the original micrograph series shown in Appendix S1). The dimensions of each image were resized to 360 × 360 pixels. Transverse end walls of the cells can be observed, for example, as the “shadows” that appear in the yellow/orange late‐maturing metaxylem vessel (LMX) 2 cells in sections 049 and 050. The color code is the same as in Figs. 5, 6, 7.

**VIDEO S1.** Animated video of the final 3D virtual reconstruction shown in Fig. 7.
