# Supplementary material for: Three‐dimensional digital image construction of metaxylem vessels in root tips of Zea mays subsp. mexicana from thin transverse sections
Source: Appl Plant Sci. 2020 May 26;8(5):e11347. doi: 10.1002/aps3.11347 (PMC7249274; doi:10.1002/aps3.11347)

Miki et al.—Applications in Plant Sciences 2020 8(5)—Data Supplement S1
DOI

APPENDIX S1. Micrographs of 127 RNase-treated, toluidine blue–stained transverse serial sections from a single teosinte root tip. Each section is 5616 × 3744 pixels, corresponding to 270 × 180 µm at magnification of 50×.

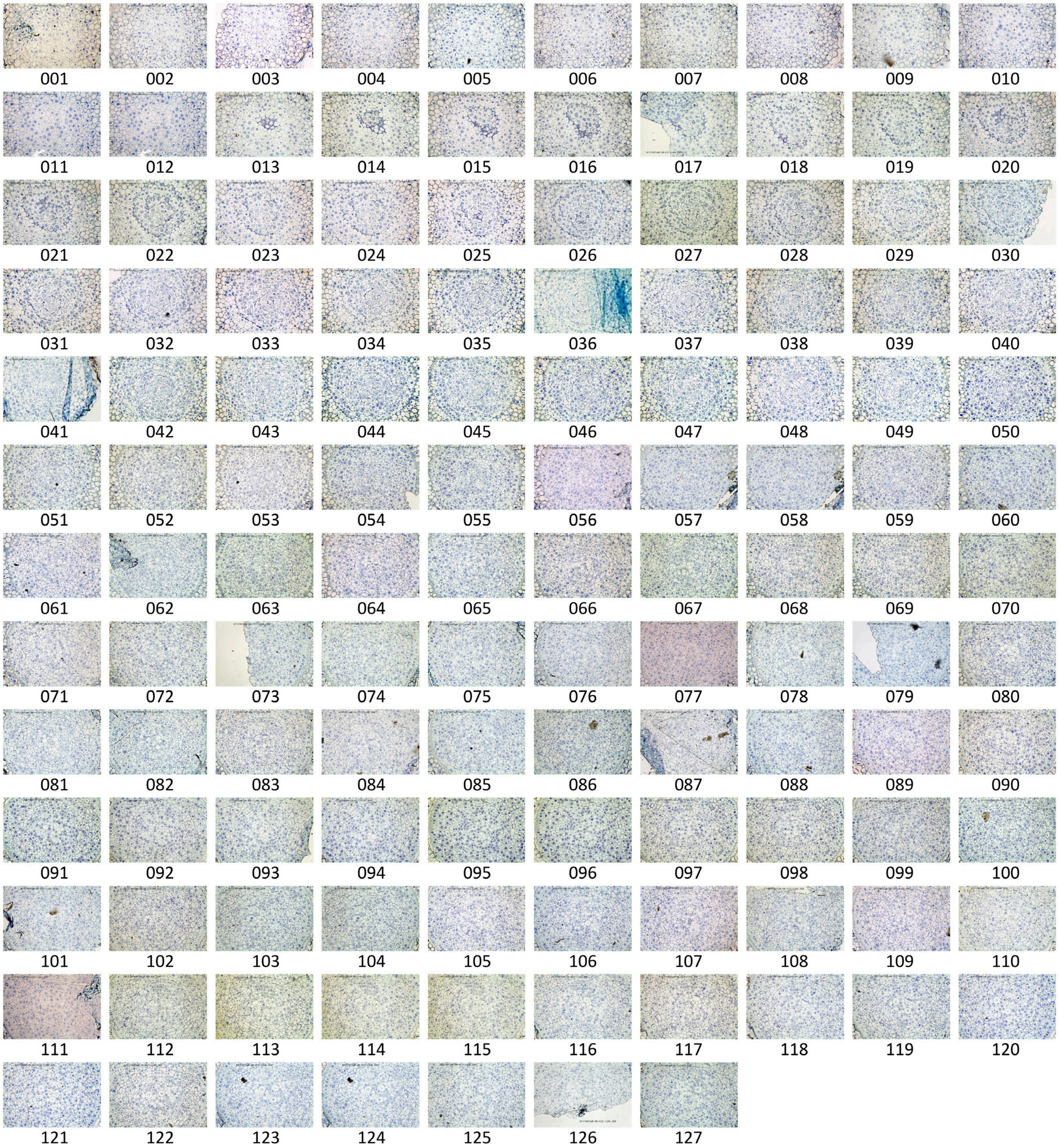

Supplement: Supplementary file 1 — APPENDIX S1. Micrographs of 127 RNase‐treated, toluidine blue–stained transverse serial sections from a single teosinte root tip. Each section is 5616 × 3744 pixels, corresponding to 270 × 180 μm at magnification of 50×. [file APS3-8-e11347-s001.pdf]
