# Supplementary material for: Three‐dimensional digital image construction of metaxylem vessels in root tips of Zea mays subsp. mexicana from thin transverse sections
Source: Appl Plant Sci. 2020 May 26;8(5):e11347. doi: 10.1002/aps3.11347 (PMC7249274; doi:10.1002/aps3.11347)

DOI

APPENDIX S2. Layers of the cuboid produced from 60 transverse sections (sections 021 to 080 of the original micrograph series shown in Appendix S1). The dimensions of each image were resized to 360 × 360 pixels. Transverse end walls of the cells can be observed, for example, as the "shadows" that appear in the yellow/orange late-maturing metaxylem vessel (LMX) 2 cells in sections 049 and 050. The color code is the same as in Figs. 5–7.

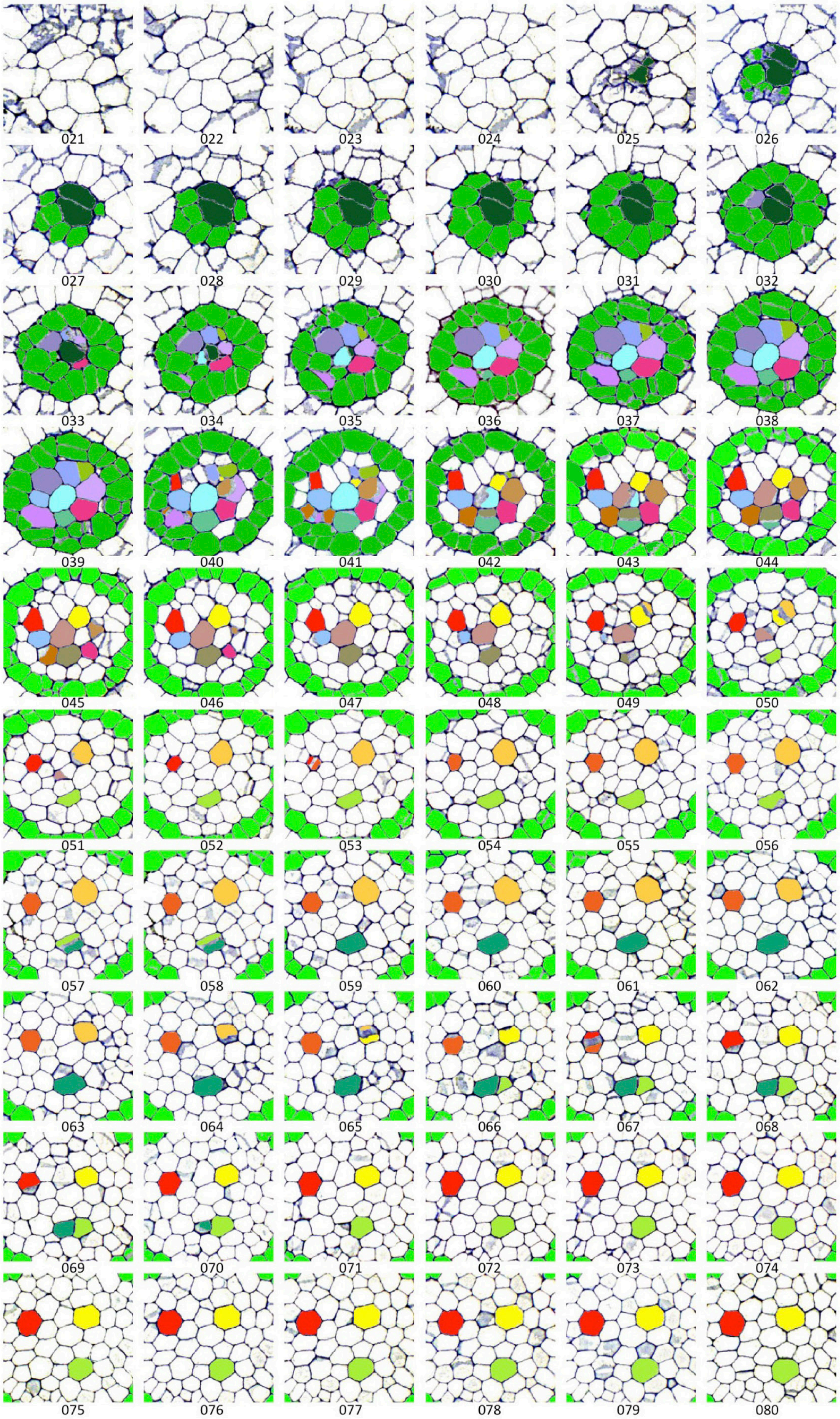

Supplement: Supplementary file 2 — APPENDIX S2. Layers of the cuboid produced from 60 transverse sections (sections 021 to 080 of the original micrograph series shown in Appendix S1). The dimensions of each image were resized to 360 × 360 pixels. Transverse end walls of the cells can be observed, for example, as the “shadows” that appear in the yellow/orange late‐maturing metaxylem vessel (LMX) 2 cells in sections 049 and 050. The color code is the same as in Figs. 5, 6, 7. [file APS3-8-e11347-s002.pdf]
